# Socio-structural and direct health challenges related to illness management among patients with type 2 diabetes in Kenya and Tanzania during the COVID-19 pandemic: A qualitative inquiry

**DOI:** 10.1371/journal.pgph.0003876

**Published:** 2025-04-09

**Authors:** Sally Mmanyi Mtenga, Irene Mashasi, Lyagamula Kisia, Peter Binyaruka, Honorati Masanja, Shukri F. Mohamed, Richard E. Sanya, Grace Mhalu, Grace Magembe, Kaushik Ramaiya, Gershim Asiki, Frances Mair, Christopher Bunn, Cindy M. Gray

**Affiliations:** 1 Health System Impact Evaluation and Policy Department, Ifakara Health Institute, Dar es Salaam, Tanzania; 2 Chronic Diseases Management Unit, African Population and Health Research Center, Nairobi Kenya; 3 Ministry of Health, Dodoma, Tanzania; 4 Shree Hindu Mandal–Hospital, Dares es Salaam, Tanzania; 5 School of Health and Wellbeing, University of Glasgow, Glasgow, United Kingdom; 6 School of Social and Political Sciences, University of Glasgow, Glasgow, United Kingdom; Federal University Birnin Kebbi, NIGERIA

## Abstract

During COVID-19, people with type 2 diabetes (T2D) experienced increased vulnerability, including severe COVID-19 complications, disruptions in diabetes management, and social isolation. These aspects were heightened in many sub-Saharan African countries, such as Kenya and Tanzania, where healthcare systems already face critical challenges in coping with increasing non-communicable diseases (NCDs). Little is known about how people with T2D in these countries managed their diabetes or how the different approaches to COVID-19 control (Kenya imposed lockdown and curfew, whereas Tanzania adopted less strict measures) impacted their T2D management. This qualitative study aimed to compare the accounts of T2D patients in both countries to examine similarities and differences in the illness management challenges they faced during the COVID-19 pandemic.Semi-structured interviews were conducted with 52 patients (Kenya, n=22; Tanzania, n=30), and the transcripts were analyzed thematically. Despite different COVID-19 control measures, patients in both countries faced similar direct health challenges, such as difficulties accessing diabetic consultations and treatment, but they also experienced distinct socio-structural challenges. Direct health challenges included difficulties in accessing diabetic consultations and treatment, limited availability of diabetic medicine at health facilities and mental health distress. These were exacerbated by socio-structural challenges, many of which pre-dated COVID-19 but intensified during the pandemic. These included closure of diabetic clinics in Dar es Salaam, business instability, financial difficulties, health insurance challenges, higher food prices impacting patients’ adherence to T2D dietary recommendations (in both countries), and price inflation of diabetic medicine and test kits (in Kenya). Together, these challenges led to patients practicing self-medication, missing doses and resulted in poor blood sugar control. People with T2D in Kenya and Tanzania have described similar illness management challenges. In both countries, future contingency planning is essential to ensure adequate routine management of T2D and to improve access to care in emergency situations. Affordable comprehensive health insurance, economic support, and psychosocial services are required to increase patient resilience and support the health and wellbeing of people with T2D.

## Introduction

The COVID-19 pandemic is one of the most devastating global health crises ever recorded, with unprecedented socioeconomic [1] and physical and mental health impacts [2,3]. The reported number of COVID-19 cases and fatalities appeared to be lower in sub-Saharan Africa than in other parts of the world. However, the socioeconomic impact of the pandemic was worse in sub Saharan Africa than in high-income countries due to pre-existing income inequalities, limited access to healthcare, and economic vulnerability [4]. The lack of adequate resources and technology for contact tracing posed significant challenges to containing the spread of COVID-19, further hindering a smooth post-pandemic recovery in sub Saharan Africa [4,5]. Furthermore, inconsistent surveillance and reporting in many sub Saharan African countries, including Kenya and Tanzania, mean that the real impact of COVID-19 may be far greater than the current estimates suggest [6].

COVID-19 emerged at a time when Kenya and Tanzania were re-organizing their healthcare systems to tackle the increasing prevalence of non-communicable diseases (NCDs) [7–12]. Type 2 diabetes (T2D) is one of the major NCDs contributing to morbidity and mortality in both countries [7–13], and its prevalence is projected to more than double by 2050 [14]. Prior to the COVID-19 pandemic, both countries experienced critical challenges in managing T2D [15,16], including the cost and access to medication and blood glucose test kits [17], which left many patients facing inadequate treatment [16]. In qualitative enquiries, people living with T2D in Kenya and Tanzania reported frequent drug shortages [18], challenges in travelling to and accessing clinics [19,20], and financial obstacles to purchasing medicines and appropriate foods [18,20,21].

Before COVID-19 outbreak, people living with T2D in Kenya and Tanzania were already facing major health challenges and suboptimal care. The situation worsened during COVID-19, when diabetic healthcare services were severely disrupted globally [22], which, together with increased stress, poor diet, and physical activity restrictions, led to greater health complications [23] and mortality [24–26] among people with T2D. In Kenya, for example, it was estimated that 67% of patients who died from COVID-19 in 2020 had underlying diabetes or hypertension [26]. Additionally, fear of social interactions weakened social support and reduced business opportunities and income for people with T2D [27,28].

A recent study in Tanzania established that the incidence of catastrophic healthcare expenditure (when out-of-pocket healthcare costs exceeded 10% of total household spending) among people with T2D increased by up to 55% [29]. One of the possible explanations for this observation is limited access to health insurance, which could provide patients with affordable health care. In both Kenya and Tanzania, there is no specific health insurance policy that responds to the needs of chronically ill people specifically those with T2D.

Kenya and Tanzania have responded differently to COVID-19. Although both countries introduced personal protective measures, including mask-wearing and handwashing/sanitising, Kenya also imposed stricter restrictions (lockdowns, curfews, and social distancing) [30,31], while Tanzania promoted the use of traditional remedies to boost immunity [31]. This contrasting response in two neighbouring sub Saharan African countries provides an opportunity to examine how the different policy contexts impacted the ability of T2D patients to manage their illness during COVID-19. Therefore, the current qualitative study aimed to explore illness management challenges experienced by patients with T2D in Kenya and Tanzania during COVID-19. These insights are crucial to inform policies related to the management of T2D and other NCDs during future pandemics or similar emergency situations in Kenya, Tanzania, and other countries in sub Saharan Africa.

## Methods

### Study design

This study was conducted in accordance with the Consolidated Criteria for Reporting Qualitative Research guidelines (COREQ) [32] (Table 1). A phenomenological design [33] was chosen to explore the lived experiences of people with T2D during COVID-19, focusing on their perceptions and interpretations of their illness management challenges, compare, and contrast the personal experiences of patients with T2D in Kenya and Tanzania during COVID-19. Face-to-face semi-structured interviews allowed participants to talk freely about managing their own illness during the pandemic and to raise any relevant issues that were important to them [34]. The interview schedule was informed by the World Health Organization (WHO) Social Determinants of Health Framework [35], which suggests that an individual’s health is influenced by their wider socioeconomic environment (e.g., income, employment, education, food security, and access to healthcare services). The schedule explored participants’ experiences of living with T2D during COVID-19, any disruption or challenges to their healthcare, and any changes in their management of T2D. It was iteratively developed by qualitative research team members from Kenya, Tanzania, and the UK, with final adjustments made following piloting during the research assistant training.

**Table 1:** COREQ Checklist.

Domain 1: Research team and reflexivity		Location in manuscript (Section, page no.)
**Personal Characteristics**		
1. Interviewer/facilitator Which author/s conducted the interview?	IM and LK	Methods - 6
2. Credentials What were the researcher’s credentials? E.g. PhD, MD	MA	Title page - 1
3. Gender Was the researcher male or female?	Females	–
4. Experience and training What experience or training did the researcher have?	At the time of the interviews, the researchers who directly conducted the interviews had already acquired qualitative skills and experience in qualitative research from other previous studies	6
**Relationship with participants**		
5. Relationship established Was a relationship established prior to study commencement?	No. The researchers were part of the team that introduced the study to the local community leaders, counties and district officials. However, none had any related affiliations that could influence the interviews	6
6. Participant knowledge of the interviewer What did the participants know about the researcher? e.g., personal goals, reasons for doing the research	Participants in Kenya and Tanzania were informed on the purpose of the study and understood that it was a research project that aimed to understand their experience in managing type 2-diabetes during COVID-19. They also understood the name of the project as GECO project. They were aware that the ethical approval had been granted. Participants reviewed the participant information in the informed consent prior to giving their written informed consent. They were also aware that participation in the study was voluntary	6, 8
7. Interviewer characteristics What characteristics were reported about the interviewer/facilitator? e.g., Bias, assumptions, reasons and interests in the research topic	No interviewer-related biases identified	Page 6
**Domain 2: study design**		
**Theoretical framework**		
8. Methodological orientation and Theory What methodological orientation was stated to underpin the study? e.g., grounded theory, discourse analysis, ethnography, phenomenology, content analysis	Ethnographic (in-depth interviews) and thematic inductive and deductive analysis	Methods – 6-7
Participant selection		
9. Sampling How participants were selected? e.g., purposive, convenience, consecutive, snowball, Purposive	In Kenya and Tanzania, patients with type 2 diabetes were recruited from the health facility patient database and registers. Health care professionals assisted in the recruitment process after having being informed about the purpose of the study	Methods - 5
10. Method of approach How were participants approached? e.g., face-to-face, telephone, mail, email	Face to face	Methods - 6
11. Sample size How many participants were in the study?	N=21 in Kenya N=30 in Tanzania	Results – 8 and Table 1
12. Non-participation How many people refused to participate or dropped out? Reasons?	Of all the participants that were invited for a semi-structured interview, all gave informed consent and completed the interview. There were no participants who refused to **participate** or complete the interviews, withdrew consent or dropped out	Methods - 6
**Setting**		
13. Setting of data collection Where was the data collected? e.g., home, clinic, workplace	The choice of setting for data collection was determined by the respondents. Some opted to be interviewed at the health facilities, others opted to be interviewed at their place of work **and** some opted to be **interviewed** at homes	Methods - 6
14. Presence of non-participants Was anyone else present besides the participants and researchers?	No	–
15. Description of sample What are the important characteristics of the sample? e.g., demographic data, date	Most respondents were middle-aged or older (95% in Kenya, 90% in Tanzania). There were slightly more women than men (55% in Kenya, 57% in Tanzania). More participants were educated to secondary school or diploma level in Kenya than in Tanzania, and in both countries, most were currently employed or self-employed	Results –Table 1
	Data were conducted between February and March 2022	
**Data collection**		
16. Interview guide Were questions, prompts, guides provided by the authors? Was it pilot tested?	Semi-structured interviews were conducted to explore **participants**’ experiences of living with T2D during COVID-19, any disruption or challenges to their healthcare, and any changes in **their** management of T2D In Kenya interviews were conducted in English or Kiswahili, according to participants’ preferences. In Tanzania, all interviews were conducted in Kiswahili	Methods - 4
17. Repeat interviews Were repeat interviews carried out? If yes, how many?	No	N/a
18. Audio/visual recording Did the research use audio or visual recording to collect the data?	The semi-structured interviews were audio recorded (face to face) using phones and or tape recorders	Methods - 7
19. Field notes Were field notes made during and/or after the interview or focus group?	Field notes were taken to remind the researchers on contextual aspects that would aid data interpretation or data collection practices that need to be shared with other team members	Methods - 6
20. Duration What was the duration of the interviews or focus group?	The semi-structured interviews lasted between 45 and 60 **minutes**	Methods - 6
21. Data saturation Was data saturation discussed?	Yes	Methods - 6
22. Transcripts returned Were transcripts returned to participants for comment and/or correction?		No
**Domain 3: analysis and findings**		
**Data analysis**		
23. Number of data coders How many data coders coded the data?	4	Methods - 6
24. Description of the coding tree Did authors provide a description of the coding tree?	Inductive and deductive codes were applied to participant’s expressions into a meaningful category Open coding was used to selects relevant narratives, and attaches a code (or codes) that reflect the aspects that are relevant to the broader research topic or research question within that data segment	Methods 6-7
25. Derivation of themes Were themes identified in advance or derived from the data?	Themes were derived from the data	Methods - 6
26. Software What software, if applicable, was used to manage the data?	Microsoft word and NVivo 12	Methods - 7
27. Participant checking Did participants provide feedback on the findings?		No
**Reporting**		
28. Quotations presented Were participant quotations presented to illustrate the themes/ findings? Was each quotation identified? e.g., participant number	Yes, most themes were supported by direct quotes attributed to anonymized Participant	Results – 8-14
29. Data and findings consistent Was there consistency between the data presented and the findings?	Yes	–
30. Clarity of major themes Were major themes clearly presented in the findings?	Yes	–
31. Clarity of minor themes	All relevant themes (main and sub-themes) are presented and discussed in the paper	8-17
32. Is there a description of diverse cases or discussion of minor themes?	Yes	15-18

### Study setting

The qualitative study was part of a larger mixed-methods research project which included a quantitative survey and qualitative interviews with patients with T2D and healthcare workers [36]. Data were collected from urban and rural areas in Kenya and Tanzania to explore any differences in views and experiences of T2D management in different settings, as well as in the two countries. In Kenya, the field sites were Nairobi and Kiambu counties (urban) and Vihiga and Nyeri counties (rural). The field sites in Tanzania were Dar es Salaam (urban) and Morogoro (rural) regions.

### Participants and recruitment

The wider project targeted men and women aged 18 or older diagnosed with T2D before March 2020 (i.e., before the WHO declared COVID-19 a pandemic) to ensure that all participants had experience of T2D management before and during the pandemic. Participants in both countries were recruited from local health facilities with the support of local healthcare providers. In Kenya, one rural facility was government-owned, and the rest were privately owned (by for-profit or faith-based organizations). In Tanzania, the health facilities included three government-owned urban hospitals, two faith-based rural hospitals, and one government-owned rural health center. Medical records were reviewed to identify eligible patients. In Kenya, a database of adults with T2D from 17 healthcare facilities was used and cross-checked against patient registers in each facility. In Tanzania, data collectors (with assistance from local healthcare providers) extracted information directly from paper-based patient registers at each health facility. Patients were excluded if they were bedridden, pregnant, breastfeeding, or were unable to provide informed consent at the time of study.

We used a two-stage process to identify participants for qualitative patient interviews. First, survey participants in both countries were asked if they were interested in taking part in a qualitative interview about their experiences of managing their T2D before and during COVID-19. Those who expressed interest were purposively selected according to their socio-demographic characteristics (age, gender, education, and occupation) to ensure representation of different views. Second, we asked local healthcare providers in Tanzania to identify additional T2D patients with other health conditions to ensure maximal variation of views.

### Data collection

All interviews were conducted between February and March 2022 by trained research assistants (N=4 in Kenya and N=6 in Tanzania). Experienced team of research assistants conducted the interviews under the supervision of two qualitative researchers with backgrounds in sociology (LK in Kenya and IM in Tanzania). The two researchers introduced the study to local community leaders, in their respective countries. However, no related affiliations could influence the interviews. Field notes were taken to aid data interpretation or highlight data collection practices that needed to be shared with other team members.

In Kenya, the interviews were conducted in English or Kiswahili, according to participants’ preferences; all interviews were conducted in Kiswahili in Tanzania. The interview setting was determined by participants and included health facilities, their workplace or their home. In Kenya, data collection was stopped once saturation of views was achieved (N=21 interviews). In Tanzania, data collection continued beyond the point of data saturation to ensure that additional participants who had already been invited to participate by healthcare providers were all included (N=30 interviews). Each interview lasted between 45 and 60 minutes.

Daily debriefing sessions were conducted with the research assistants to reflect on the interviews and to share ideas that would be useful for subsequent interviews. All interviews were audio-recorded (face-to-face) using phone or tape recorders with participant consent. In Tanzania, the audio-recordings were transcribed verbatim by the research assistants who conducted the interviews and reviewed by IM and SMt. In Kenya, transcription was performed using an independent transcriber and reviewed by the research assistants and LK.

### Data management and analysis

Transcripts were quality assured by field supervisors against the original audio recordings. LK and SFM (Kenya) and IM and SMt (Tanzania) read all transcripts repeatedly line by line to familiarize themselves with the data and to inductively identify themes and categories that reflected the participants’ experience of managing their T2D during COVID-19. In Kenya, transcripts were translated into English, where required, and coded using NVivo 12 software. In Tanzania, word tables were used for the preliminary coding of Kiswahili transcripts before full coding. To enhance analytic validity, a subsample of the transcripts (N=5) from each country (all translated into English) was reviewed by CMG and CB from the University of Glasgow, and two online meetings were held to allow all qualitative team members to provide feedback on an initial coding frame that had been developed by researchers from Kenya and Tanzania. Although there was good agreement overall, some adjustments of the coding frame were undertaken to allow a more accurate representation of participants’ views.

LK, SFM, IM and SMt applied the coding frame to all transcripts. Broad themes were generated and categorized deductively into two domains (socio-structural and direct health challenges), which provided a framework for comparing the similarities and differences between the two countries and between rural and urban areas. SMt then developed an initial conceptual model of T2D management challenges during COVID 19 that was shared (with related extracts) with the other research scientists (LK, SFM, IM) from Kenya and Tanzania, and with CMG and FM from University of Glasgow. The model was then adjusted according to feedback. Interview extracts with identifiers (Gender_Participant ID_Urban/Rural_Country) are provided to support the findings presented below.

### Ethics statement

In Kenya, ethical approval was obtained from the African Population and Health Research Center Ethics Review Committee (Ref. no: DOR 2021/041), the African Medical and Research Foundation Health Africa’s Ethics and Scientific Research Committee (ref. no: AMREF-ESRC P900/2020), and the National Commission for Science, Technology, and Innovation (licence no: NACOSTI/p22/14986). In Tanzania, approval was obtained from the Ifakara Health Institute (IHI/IRB/No: 38-2021) and the National Institute of Medical Research (NIMR/HQ/R.8a/Vol. X/3806). Written informed consent was obtained from all participants before their participation in an interview. Participants were aware that their participation in the study was voluntary.

## Results

### Participants

Most interviewees were of the age 60 to 81 (59% in Kenya and 67% in Tanzania). There were slightly more women than men (55% and 57% in Kenya and Tanzania, respectively). More participants were educated to secondary school or diploma levels in Kenya than in Tanzania, and in both countries, most were currently employed or self-employed (Table 2).

**Table 2:** Socio-demographic characteristics of T2D patients who participated in the study.

Characteristics	Categories	Kenya (n=21)		Tanzania (n=30)	
**n**	**%**	**n**	**%**
**Age**	33–40	1	5%	3	10%
	41–59	6	36%	7	23%
	60–81	14	59%	20	67%
**Gender**	Female	11	55%	17	57%
	Male	10	45%	13	43%
**Education**	Primary school	9	41%	19	63%
	Secondary school	9	41%	7	23%
	Tertiary	3	18%	4	13%
**Occupation**	Farmers	6	27%	10	33%
	Retired	3	9%	0	0%
	Employed	9	64%	19	63%
	Unemployed	3	0%	1	3%

### Challenges of managing T2D during COVID-19

**Fig 1:**
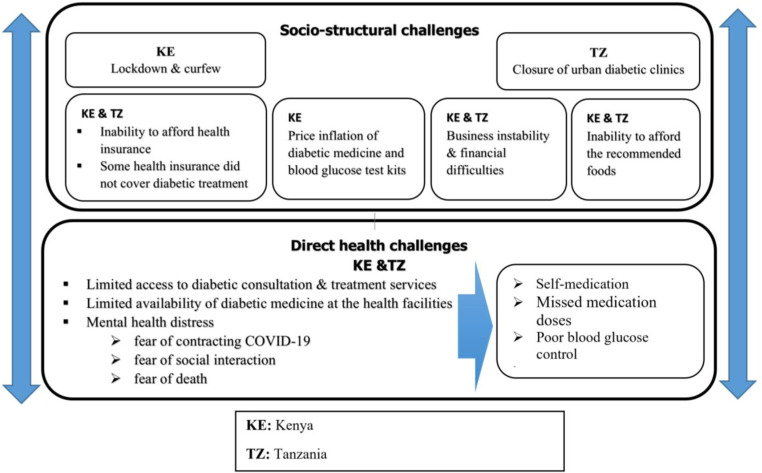
Model of the interrelated socio-structural and direct health challenges for T2D management during COVID-19.

Fig 1 provides a summary model of the challenges and impacts described by participants in Kenya and Tanzania as they navigated the management of their T2D during COVID-19. We categorized the key themes into socio-structural and direct health challenges.

The model shows that both countries initially implemented distinct measures to mitigate the pandemic, such as socio-structural measures that included lockdown and curfew (in Kenya), and closure of diabetic clinics (in Tanzania). However, patients also reported additional socio-structural factors, some preexisting but exacerbated by the pandemic, which posed challenges to their illness management. These factors included the inflation of prices for diabetic medicine and blood glucose test kits in Kenya and elevated food prices coupled with certain health insurance policies not covering diabetic treatment in both countries. Many patients have also reported high levels of business instability during the pandemic. These socio-structural challenges contribute to the direct health challenges faced by patients in both countries, including difficulties in accessing diabetic health facilities and treatment services, difficulties in accessing diabetes medication, failure to comply with dietary recommendations, poorly controlled blood sugar, and mental health distress.

Since the challenges of managing diabetes during COVID-19 are interrelated, we present the findings below with direct health challenges as key themes and the socio-structural challenges to support or explain direct health challenges. To differentiate these themes, we bolded the socio-structural challenges/factors.

#### Limited access to diabetic consultation and treatment services.

**The imposition of lockdowns and curfews** in Kenya, alongside the **closure of diabetes clinics** in Dar es Salaam, Tanzania, which were repurposed to cater to COVID-19 patients, resulted in restricted access to health facilities for diabetic consultation and treatment services. Participants’ narratives in both countries suggested that these measures, implemented to curb the spread of COVID-19, lacked alternative health system arrangements to ensure continuity of care for individuals with Type 2 Diabetes (T2D). In Tanzania, urban patients unable to access their normal diabetes clinics were directed to other health facilities. However, these facilities often do not provide diabetes services. One man described moving from one facility to another to search for treatment in vain:

*“After closing the (diabetes) clinic, they told us to shift to ZZZ (a health facility), unfortunately, we went to ZZZ and we were told there was no clinic set up yet for diabetic patients….: Of course there were others who said they went to the YYY (another health facility), but I went to WWW (another health facility) as well and I was told there was no such diabetic unit.”* [Male_18_Urban, Tanzania].

As most diabetes clinics in Tanzania are located in urban areas, accessing appropriate care was already difficult for rural patients pre-COVID and, for some, appeared to become more so during the pandemic. The inability to obtain treatment services meant that participants in both countries found themselves having to buy their diabetes medication from local pharmacies without prescription.

A participant from an urban area of Kenya described how lockdown and curfews contributed to ‘self-medication,’ which had become the new norm for some people. Two years after the outbreak of the pandemic (when the interviews were conducted), he confessed that he was still buying most of his diabetes medications directly from the local pharmacy without a prescription:

*“It was tragic because every movement was a problem; you could not leave the estate (home area) from here to where you get your medications. Even getting doctors themselves and taking medication was a problem. However, that is the time we can now rush to the nearest pharmacist and buy medicine over the counter without the doctor’s instructions. However, before (COVID-19), we could not do that because we could follow what the doctors were telling us. Therefore, it was a bad experience, and this experience introduced us to go over the counter to buy these medications. The truth is we have adapted that whenever I feel I need medication mostly we don’t go to the medics [doctors], we rush to the pharmacist.”* [Male_ 1107_Urban, Kenya].

#### Limited availability of diabetes medicine at the health facilities.

Even when patients in both countries were able to see a doctor, they complained that COVID-related supply issues meant that the medicines they needed forT2D were not always available.

A man from rural Tanzania described this as the main challenge he faced in managing his illness during the pandemic.

*“You find the doctor prescribes my diabetes medicine and when I go to the pharmacy they tell me that there is no medicine. […] Honestly, during that time (COVID-19) the challenge was the shortage of diabetes medicines at the health facility.”* [Male_14_Rural, Tanzania].

Difficulties in accessing medication led to instances in which patients had to miss doses. This was exacerbated by **business instability and financial hardships** in both the countries. Urban and rural interviewees described being unable to run businesses or work effectively during the pandemic. The fear of contracting COVID-19 prompted them to minimize social interaction, resultinginteraction, resulting in diminished business opportunities. This led to increased financial difficulties, as the income they were able to make was insufficient to meet their needs. One urban woman described how these circumstances exacerbated her challenges in affording diabetes medication, ultimately adversely affecting her health.

*“To speak the truth, before COVID-19 the economic status was good, so I was able to manage it (T2D), I was able to get medicine in time. I was doing well. However, during the COVID-19 pandemic, the economy became difficult, there was no business, and there were many challenges. I stayed for a week without money to buy the medicine. So by the time I get the medicine I start to face a challenge [..], my mouth dries, I could not sleep.”* [Female_ 12_Urban, Tanzania].

Access to medication was also hampered by **preexisting health insurance challenges**. Kenya and Tanzania have several national and private health insurance schemes available to support healthcare access. However, not everyone can afford health insurance, and around one-third of T2D patients in both countries are uninsured [26]. Interviewees without health insurance felt that this affected their ability to access the diabetic medication they needed during the COVID-19 pandemic.

*“I don’t have health insurance. If I had the insurance, it would have been easier; I could just come to the hospital and given medication. But now I need to buy (medicine) and the economic situation is not good with this corona (COVID-19) at hand. If I had money I would buy medicines to take me for two weeks or a month, and I would not miss any dose but now I can’t.”* [Female_03_Urban. Tanzania]

Patients with health insurance in both countries also reported difficulties accessing diabetes medication. This has led some to question the value of being insured. One woman from a rural area of Kenya told how she felt she was continually paying out money and getting very little in return:

*“(…) I wonder what the benefit of NHIF [national health insurance] is to me. It doesn’t help me. This is the time [during COVID-19] that I needed to use [national health insurance). When I go there [to the health facility], I fill out the form, but they do not have the medicine. I go to [the health facility]; they do not give medicine with the card. Therefore, they simply took money. I am about to stop paying for it because people are saying that if you go and get admitted, there is a money limit, and you must pay for the rest. Why do I have to keep paying for it all the time.”* [Female_1108_Rural, Kenya].

In Kenya, the problem of accessing diabetes medication was compounded by **the rapid price increases in diabetes medication and blood glucose test kits** during the pandemic. A woman from an urban area described having to use painkillers to manage her T2D since she could no longer afford the specialist medicines she should have been using:

*“Before COVID-19 the medicines were not very expensive like right now [during COVID-19]. Glycomet 850 mg [anti-hyperglycemic tablet] is ksh.10 per tablet and Novolog [rapid-acting insulin injection] is ksh.5 and I take them twice a day. So, per day, I use thirty shillings, so I have to take painkillers to manage this disease. So, this is a lot of money per day for someone who is not working and doesn’t have any support.”* [Female_1516_Urban, Kenya].

The challenges patients experienced in managing their diabetes because of rising prices and reduced income during the COVID-19 pandemic were not limited to medication. Individuals with T2D are advised to have a diet low in starch and carbohydrates to control their blood sugar levels. These foods can be expensive and become even more common during the COVID-19 pandemic. Patients in both countries, but particularly in urban areas, reported that their **inability to afford the foods recommended for T2D management** compromised their ability to manage their illness well:

*“During that time (COVID), it was economically difficulty. Sometimes I found I don’t have the food that I was told to be eating, then I get confused and in that situation, if get biryani (spiced rice) I eat, and when I finish eating, I just drink a lot of water (to dilute the carbohydrate).”* [Female_16 Urban, Tanzania].

Some urban participants further described how limitations in the food they could afford directly compromised their health.

*“During that time (COVID-19) getting food was a problem, like now I am diabetic, which is also a problem. If it is cooking oil, we are told that we should use Elianto or Rina, and getting this is a problem (expensive). If you use the other brands of cooking oil the blood sugar level will go up.”* [Female_1558_Urban, Kenya].

#### Mental health distress.

The three interrelated dimensions of fear, fear of contracting COVID-19, which resulted in fear of social interaction and fear of death, led to participants experiencing mental health distress. Almost all patients in both countries described how their anxiety about contracting COVID-19 was heightened because their healthcare providers and/or government messaging clarified that their diabetic status could increase their risk of complications.

*“I knew my condition and I was very worried thinking of my disease. I have diabetes and ulcers, so if I got COVID, what would happen to me? (..). I knew once I got COVID-19, and I was already in that special group where when you get COVID-19 infection, your body’s immune system will be low. Will I be able to survive if my immune system is compromised and I contract COVID? Regardless of whether everyone was afraid of COVID, I was the most concerned, and I know it wasn’t just me but other people with diabetes as well.”* [Female_04_Urban, Tanzania].

Despite taking extra precautions, several patients in both countries contracted COVID-19 and experienced complications, including poorly controlled blood sugar levels. One man from an urban area in Kenya described how restricted access to healthcare compounded the stress he experienced during that time:

*“Yes, I had fear when I had that attack (COVID-19) since my blood sugar levels and (blood) pressure were going up, I wondered if we will get a doctor or not because it was almost their closing time that is 3 pm.”* [Male_1315_Urban, Kenya].

Another urban participant from Tanzania said that the health complications he experienced from contracting COVID-19 contributed to the financial difficulties he faced by limiting his capacity to work:

*“I was definitely affected (by COVID-19) there are some days that my blood sugar went up, I started being sick and I could not even go to work, because I felt weak.*” [Male_ 19_Urban, Tanzania].

The fear of contracting COVID-19 also directly led to fear of social interaction. As described above, this meant that the patients were reluctant to mix with other people for work, leisure, and other activities. One patient from an urban setting described how the fear of other people stopped him from attending his regular diabetes clinic:

*“This (health facility) was chosen to manage COVID-19. So COVID patients are coming here. Myself I was avoiding gatherings (coming here) because I am already affected with diabetes and I heard that if you are diabetic and if you contract COVID you will have difficulties in recovering.”* [Male_09 _Urban, Tanzania]

Finally, for some interviewees, mental health distress was heightened because they were aware of people with diabetes or other chronic illnesses who had died because of contracting COVID-19. One woman from an urban area of Kenya explained how this meant fear of dying became very real:

*“It is a challenge since you hear of a diabetic person who has died, then another one and after a short period a patient with both diabetes and hypertension or diabetes only got COVID and they also died. I was seeing it as if I was the next one. [...] I was not okay, I had a lot of fear.”* [Female_1516_Urban, Kenya].

## Discussion

Our study shows that people living with T2D in Kenya and Tanzania experienced similar illness management challenges during COVID-19 despite the two countries employing different approaches to control the pandemic (lockdown and curfew in Kenya and closure of diabetic clinics in urban areas of Tanzania). Patients’ narratives revealed that when COVID-19 measures were imposed, there were no specific arrangements to ensure the continuity of healthcare. This led to direct health challenges, including limited access to diabetes consultations and treatment, limited availability of diabetes medicines, missed treatment, self-medication, and mental health distress (fear of contracting COVID-19, fear of social interaction, and fear of death), which patients’ accounts suggested led to problems controlling their blood sugar. These direct health challenges, which were reported by patients in urban areas and, to some extent, rural areas, were exacerbated by additional socio-structural challenges. Our study shows that many of the socio-structural factors that existed before COVID-19, such as price inflation of diabetic medicine and blood glucose test kits, business instability and financial difficulties, and inability to afford recommended foods, were heightened during the pandemic, presenting additional difficulties for patients in managing their diabetes and making them even more vulnerable to COVID-19 than they otherwise might have been.

The reported association between lockdown and healthcare disruption aligns with the evidence from other LMIC countries. For example, online surveys have demonstrated that lockdowns led to difficulties for patients in accessing routine consultations and testing in Indonesia and Pakistan [37,38]. A qualitative study in Uganda revealed that curfews (and the resulting travel restrictions) and fear of contracting COVID-19 were important factors in preventing patients from attending routine healthcare visits [39]. Furthermore, in South Africa, retrospective testing of blood samples from T2D patients showed a direct link between lockdown and poor diabetes control [40]. Although Tanzania did not impose strict COVID-19 measures, the closure of diabetic clinics in Dar es Salaam also disrupted patients’ access to routine T2D care, as they did not receive formal advice about alternative ways of accessing treatment. Therefore, our findings underline the importance of advanced contingency planning to ensure that during future pandemics or other similar emergency situations, formal arrangements to ensure continued provision of routine healthcare are made and clearly communicated to T2D patients to reduce their risk of experiencing complications.

Patients in both countries reported practicing self-medication (buying diabetes medicines from the pharmacy without consulting a healthcare practitioner) as an illness management strategy during the COVID-19 pandemic. For some patients this behaviour has persisted. It remains unclear how many patients with T2D are yet to resume routine health checkups, but it may be important for the ministries of health in both countries to promote the need for regular medical checkups to prevent health complications including comorbidities [41,42].

Patients in rural and urban areas in both countries also reported problems accessing diabetes medication during COVID-19. While concerns about the poor availability of medicines are not new, it is clear that global medical supply chains were severely impacted during the pandemic [43]. This is likely to have contributed to the higher cost of diabetic medicine reported by patients in Kenya. This contention is supported by a qualitative study of healthcare professionals in Ghana that reported higher medicine and healthcare costs during the COVID-19 pandemic [44]. It is less clear why patients in Tanzania did not directly report higher medication costs; however, many complained that increased business instability and insufficient income made it difficult for them to afford diabetes medication and treatment. Studies in South Africa [45], Bangladesh [46] and India [47] similarly reported job losses, reduced income, and reduced business activity among patients with long-term conditions. Experiencing financial problems meant that some patients in both Kenya and Tanzania were unable to afford the recommended diet for T2D management. This was worsened by higher food prices across the sub Saharan African region [48], which was exacerbated by widespread global supply chain disruption [43]. The experience of patients in LMICs contrasts with that of patients in higher-income countries, where governments employ economic support measures (e.g., furlough) to help people cope financially during the pandemic. Therefore, other measures that could be considered during future pandemics and emergency situations to reduce the health risk to vulnerable populations, such as people with T2D, include regulation/subsidies of essential medication and food.

Although T2D patients have long experienced health insurance challenges in both Kenya and Tanzania [49], our study suggests that these challenges contributed to the direct health challenges they reported during COVID-19. This is an important observation, as achieving universal health coverage (as highlighted by the United Nations Sustainable Development Goals) is critical for patients with chronic illnesses (including T2D) whose healthcare needs may make them particularly vulnerable to financial hardship during pandemics [29]. A health insurance system that is economically resilient [50], with transparent and fully accountable governance systems, is essential to meet the healthcare needs of all, including during and after, emergency situations such as COVID-19 [51].

Finally, mental health distress emerged as an important issue in this study. A key contributory factor to this mental health distress was the fear of contracting COVID-19, which was compounded by the inability to access healthcare services. Similar findings were reported by an online survey in Egypt, showing that depression, anxiety, and stress among patients with chronic diseases during COVID-19 were associated with reduced use of routine healthcare and treatment services [52]. COVID-related anxiety also led to a fear of social interaction, which other studies have shown to compound mental health distress. For example, a qualitative study in Uganda revealed that not being able to see relatives and friends during COVID-19 contributed to increased depression and stress among chronically ill patients [39]. A narrative review of the impact of COVID-19 on healthcare revealed that patients with chronic illness who were socially isolated were more likely to experience psychological problems than those with good social support [53].

### Strengths and limitations

To the best of our knowledge, this qualitative study is the first to explore the illness management challenges experienced by T2D patients in Kenya and Tanzania during the COVID-19 pandemic. One important strength is the focus on two neighboring countries with differing socio-structural responses to COVID-19, which enhances the internal validity of our study. The inclusion of both rural and urban settings in both countries facilitated a nuanced understanding of the impact of local contextual factors on patient experiences, thereby enriching the findings. However, the narrow geographic scope, particularly in Tanzania, where patients were sampled from health facilities in only two areas, may limit the generalizability of the findings. This could potentially restrict the applicability of the study’s conclusions to a broader population. Additionally, as with any qualitative research, the interpretation of data is subjective and influenced by researchers’ perspectives, which is likely to introduce bias into the findings.

Furthermore, since the interviews relied on patients’ subjective experiences, this may attract recall or social desirability bias. Nonetheless, efforts have been made to mitigate bias through rigorous data analysis procedures and the involvement of multiple researchers in the interpretation process.

## Conclusions

Our study highlights that individuals living with T2D in two sub-Saharan African countries, despite facing distinct approaches to COVID-19 control, encountered similar challenges in managing their illness. Having patients experiencing similar level challenges could indicate that chronically ill patients may be sharing similar patterns of vulnerabilities and challenges pertaining to their illness management across localities. The common vulnerability may include heightened anxiety regarding their health risks. Our findings highlight the need for targeted interventions to meet the healthcare, financial, and psychological needs of T2D patients. There is a need to ensure the provision of affordable comprehensive health insurance, economic support, and psychosocial services to help patients overcome mental distress. In addition, advance contingency planning is essential to ensure continued access to routine diabetic consultation and treatment services during future pandemics or emergency situations. Furthermore, it is important to provide information on how to access such alternative services and the importance of having regular medical checkups must be clearly communicated to T2D patients.

Researchers have highlighted that, ‘if there is one thing this pandemic has shown us, it is that diabetes care needs to be a central component of the planning for the next emergency’ [54]. With the number of cases of diabetes in Africa projected to reach 55 million by 2045 [55], this is an urgent priority.

Concerted efforts are needed to integrate diabetes care into emergency preparedness strategies to ensure the resilience of healthcare systems to effectively manage such crises in the future.

Future studies may explore more about how the emergency response programs at the primary health care can be improved to respond to the needs of patients with type 2 diabetes and other chronically illness patients during pandemic. In addition, studies on health insurance need to investigate on how the health insurance can be made affordable to patients with type 2 diabetes and those with chronic illness. In addition, further studies are required to understand the challenges experienced by other patients with chronic illness to establish a comprehensive understanding about the challenges faced by patients with chronic illness.

## Supporting information

S1 Checklist xxx. (RTF)
